# Unraveling the Beneficial Role of Resveratrol in Fructose-Induced Non-Alcoholic Steatohepatitis with a Focus on the AMPK/Nrf2 Signaling Axis

**DOI:** 10.3390/medicina61010139

**Published:** 2025-01-16

**Authors:** Soha S. Zakaria, Safaa M. Hanafy

**Affiliations:** 1 Department of Biochemistry, College of Medicine, Imam Mohammad Ibn Saud Islamic University (IMSIU), Riyadh 13317, Saudi Arabia; 2 Department of Anatomy and Physiology, College of Medicine, Imam Mohammad Ibn Saud Islamic University (IMSIU), Riyadh 13317, Saudi Arabia; aabdelaziz@imamu.edu.sa

**Keywords:** non-alcoholic steatohepatitis (NASH), resveratrol, albino rats, oxidative stress

## Abstract

*Background and Objectives*: High fructose intake is associated with non-alcoholic fatty liver disease (NAFLD), a chronic liver disease that is on the rise worldwide. New alternatives for treatment, such as bioactive phytochemicals, are needed. The aim of this study was to investigate the beneficial role of resveratrol in treating non-alcoholic steatohepatitis (NASH). *Materials and Methods*: Sixty male albino rats were allocated to three groups: group I, the normal control group; group II, the fructose-enriched diet group (FED), which was fed a 70% fructose diet for six weeks to induce NASH; and group III, the resveratrol–FED group (RES + FED), which was given the same FED diet plus an oral dose of 70 mg/kg resveratrol (RES) every day for an additional six weeks. We performed histological evaluations and assessed blood lipids and liver enzymes to study resveratrol’s impact on NASH. Quantitative real-time PCR was used to assess the mRNA expression of nuclear factor E2-related factor 2 (Nrf2) in the liver samples. ELISA was used to measure Beclin 1, AMPK, IL-6, and the DNA-binding activity of Nrf2. Oxidative stress indicators, including GSH, SOD, and MDA, were evaluated spectrophotometrically. *Results*: Resveratrol effectively alleviated the biochemical and histopathological abnormalities associated with NASH, improving autophagy by raising Beclin 1 levels while reducing inflammation by decreasing IL-6 levels. Furthermore, resveratrol restored the liver architecture and the oxidative balance, as evidenced by the decreased MDA levels and improved antioxidant status via elevated GSH and SOD activities, as well as the activation of the AMPK/Nrf2 signaling axis. *Conclusions:* This study specifically examines resveratrol’s therapeutic effects in a high-fructose diet-induced NASH model, focusing on the AMPK/Nrf2 signaling pathway to address oxidative stress and autophagy, providing novel insights into its molecular mechanism of action. Resveratrol reduces NASH by boosting autophagy and activating the AMPK/Nrf2 pathway. These findings underscore the potential of resveratrol as a promising therapeutic agent that can support treatment alongside conventional medications in the management of non-alcoholic steatohepatitis (NASH).

## 1. Introduction

Globally, non-alcoholic fatty liver disease (NAFLD) is the most prevalent liver disease, more recently known as metabolic dysfunction-associated steatotic liver disease (MASLD). Up to 30% of adults in Western nations and 15% in Asian nations are impacted, and the number of affected children is rising [1]. Triacylglycerol that accumulates intrahepatically and exceeds 5% of liver weight is a hallmark of non-alcoholic fatty liver disease (NAFLD). NAFLD spans a range of conditions, from triacylglycerol buildup and steatosis to non-alcoholic steatohepatitis (NASH), an inflammatory reaction that can lead to liver cirrhosis and a higher risk of hepatocellular cancer [2]. Central obesity, insulin resistance, fasting hyperglycemia, and hypertriglyceridemia are the most likely risk factors for NAFLD/NASH [3]. According to the “two-hit” theory, patients with simple steatosis develop NASH because liver steatosis makes hepatocytes more vulnerable to second hits, which include oxidative stress, mitochondrial dysfunction, and cytokine/adipokine imbalance, which can result in inflammation and fibrosis [4].

Dietary consumption of fructose has dramatically grown. Consuming large amounts of fructose can harm the liver by affecting the metabolism of cholesterol, triacylglycerols, and carbohydrates. Moreover, it raises inflammation and advances NAFLD [5,6].

Lysosomal degradation eliminates damaged cellular components via autophagy. Approximately 30 mammalian proteins are involved in the formation of the isolation membrane. Beclin 1, a key protein, interacts with Bcl-2 and is crucial for starting autophagy. Its induction is regulated by the AKT/mTOR and AMPK pathways [7,8,9]. The verified functions of autophagy in the metabolism of lipids, the sensitivity of insulin, and cell damage indicate its roles in NASH [10]. The AMP-activated protein kinase (AMPK) protein is made of non-catalytic β- and γ-subunits and a catalytic α-subunit and is implicated in energy homeostasis in eukaryotic cells. AMPK is strongly controlled by various regulatory proteins, including mTORC1, which is phosphorylated and inhibited when AMPK is activated [11].

Hepatocytes have cytoprotective enzymes that protect against toxins. Genes containing antioxidant response elements (AREs) in their promoters encode these enzymes; their transcription is based on cellular redox changes [12]. Nuclear factor-erythroid 2-related factor 2 (Nrf2) is a basic leucine zipper transcription factor that controls the transcriptional induction of genes containing AREs that encode heat-shock proteins, electrophile-conjugating enzymes, ubiquitin/proteasomes, and antioxidant enzymes in response to cellular stressors such as reactive oxygen species (ROS) [13,14]. Prior studies suggest the importance of targeting oxidative stress and autophagy in managing NASH [15,16]. However, there is limited research exploring how the AMPK/Nrf2 pathway is modulated in high-fructose-induced NASH, creating a significant research gap that this study aims to fill.

Resveratrol is a phenolic molecule that belongs to the stilbene family of phenols and is widespread in plants, including white tea, cassia seeds, and grape shells, with its primary source being Polygonum multiflorum rhizome extract. The protective effects of resveratrol involve the regulation of multiple signaling pathways, including the inhibition of oxidative stress and inflammation, the enhancement of insulin sensitivity, the induction of autophagy, the regulation of lipid metabolism, the promotion of GLUT4 expression, and the translocation and activation of the SIRT1/AMPK signaling axis. While resveratrol has been extensively studied for its general antioxidant, anticancer, and anti-inflammatory properties, its effects on AMPK/Nrf2 signaling in the context of a high-fructose diet remain underexplored [15,16]. Therefore, the objective of this study was to assess the beneficial effects of resveratrol on biochemical and histopathological changes related to high-fructose diet-induced NASH to provide a new understanding of the regulatory function of resveratrol in liver lipid metabolism, positioning it as a promising candidate for reducing NASH. Additionally, this study aimed to elucidate the function of AMP/Nrf2 signaling and autophagy in the development of steatohepatitis and relate them to oxidative stress indicators.

## 2. Materials and Methods

### 2.1. Chemicals

Most of the chemicals, including resveratrol and D-fructose (purity: ≥99%), were acquired from Sigma-Aldrich compounds (St. Louis, MO, USA).

### 2.2. Animals

#### 2.2.1. Animals and Diets Used in the Experiment

This study included 60 male albino rats weighing between 185 and 200 g, acquired from the animal house at Tanta University’s Faculty of Science, Egypt. The animals were kept in controlled environments with a 12 h light/dark cycle, a constant temperature of 25 °C ± 2 °C, and a humidity of 60% ± 10%. Water and a standard chow meal were provided freely for two weeks before the start of the experiment. The experiments and animal care were carried out in compliance with the guidelines of Tanta University’s Research Ethics Committee (approval code: 2806/1/22).

#### 2.2.2. Animal Grouping and Experimental Design

Following the adaptation phase, rats were randomly assigned to three equally sized groups: Group I (the control group) received a standard caloric diet (composed of 59.7% carbohydrates, 10.6% fat, and 27.3% protein) and had free access to plain water. Group II (the FED group) was given a fructose-enriched diet along with a standard caloric diet and 70% fructose-sweetened water (*w*/*v*) for 6 weeks to promote NASH [17]. Group III (the RES + FED group) was provided with the same standard caloric diet and 70% fructose-sweetened water (*w*/*v*) for 6 weeks, like group II, and then received daily oral administration of resveratrol (RES) via gavage at a dosage of 70 mg/kg, suspended in 0.5% carboxymethyl cellulose (CMC), for an additional 6 weeks [18].

Concurrently, equal amounts of CMC were given intragastrically to the control group for 6 weeks. The dosage was modified weekly to account for variations in the rat’s body weight to ensure the dosage per kilogram of body weight remained consistent throughout this study.

### 2.3. Blood and Tissue Sampling

#### 2.3.1. Blood Sampling

Upon the completion of the experiment, the animals were fasted for 18 h to reduce feeding-induced changes in lipid patterns and were then decapitated while under anesthesia. Serum was extracted by centrifuging blood in a sterile, dry tube at 3000× *g* for 20 min at 4 °C. The tube was then stored at −70 °C until analysis.

#### 2.3.2. Tissue Sampling

After being removed, the liver was perfused in situ with cold 0.9% (*w*/*v*) NaCl solution, dried on filter paper, and separated into two pieces. One portion was kept in 10% buffered paraformaldehyde for histological analysis. The remaining portion was kept at −70 °C for tissue homogenate preparation and gene expression analysis.

### 2.4. Liver Tissue Homogenate and Nuclear Extract Preparation

One piece of each specimen was weighed and homogenized in ice-cold 10 mM potassium phosphate with 1 mM EDTA (pH 7.4) at a ratio of 1/5 *w*/*v*, using a Potter–Elvenhjem tissue homogenizer(Bellco Glass, Inc., located in Vineland, NJ, USA). The homogenates were centrifuged at 12,000× *g* for 30 min at 4 °C. The supernatant was then stored at −70 °C for further analysis. The Nuclear/Cytosol Fractionation Kit (Bio Vision, Milpitas, CA, USA) was used to produce the nuclear extract of liver cells. The samples’ total protein content was determined using standard bovine serum albumin (Bio-Rad Protein Assay) according to the Bradford technique [19].

### 2.5. Biochemical Analysis

#### 2.5.1. Lipid Analysis

Enzymatic–colorimetric techniques were used to test triacylglycerols (TAGs) and total cholesterol (TC) (Bio diagnostic, Giza, Egypt).

#### 2.5.2. Liver Function Tests

Serum alanine transaminase (ALT) and aspartate aminotransferase (AST) levels were measured using Randox kits(Randox Laboratories Ltd., Crumlin, Northern Ireland, UK).

#### 2.5.3. Oxidative Stress Parameters

Hepatic reduced glutathione (GSH) [20], superoxide dismutase (SOD) [21], and malondialdehyde (MDA) [22] levels were measured using spectrophotometric assays using commercial kits (Bio-diagnostic, Giza, Egypt).

#### 2.5.4. Adenosine Monophosphate Protein Kinase (AMPK) Levels

An ELISA kit from Glory, Lisle, IL, USA, was used to test the active phosphorylated form of AMPK.

#### 2.5.5. Hepatic Beclin 1 Levels

Hepatic Beclin 1 levels were measured using an ELISA kit from Glory, USA.

#### 2.5.6. Hepatic IL-6 Levels

Hepatic IL-6 levels were assayed using the Rat IL-6 ELISA Kit (Thermo fisher scientific Cat #ERA31RB, Thermo fisher, Waltham, MA, USA).

#### 2.5.7. Hepatic Nuclear Factor Erythroid 2-Related Factor 2 (Nrf-2) DNA-Binding Activity

Hepatic nuclear factor erythroid 2-related factor 2 (Nrf-2) DNA-binding activity was analyzed in liver nuclear extracts using an ELISA kit (Cayman, Ann Arbor, MI, USA).

#### 2.5.8. Nuclear Factor-Erythroid 2-Related Factor-2 (Nrf2) Gene Expression Measurement

Real-time PCR was used to determine Nrf2 gene expression in liver tissue (RT-PCR). The Gene JET RNA Purification Kit (Thermo Scientific, Waltham, MA, USA) was used to isolate total RNA from liver tissues. Aid H Minus Reverse Transcriptase (Thermo Scientific, USA) was used to reverse transcribe total RNA and create cDNA, which was then employed as a template. The reaction was carried out using a Power SYBR Green PCR Master Mix (Life Technologies, Carlsbad, CA, USA). Then, the cDNA was amplified using the Step One device (Applied Biosystems, Waltham, MA, USA), followed by 5 min of initial denaturation at 95 °C, thirty cycles of denaturation at 95 °C for 30 s, annealing at 60 °C for 30 s, and extension at 72 °C for 30 s. A control reaction without a DNA template was conducted concurrently to detect genomic DNA contamination. The housekeeping gene, b-actin, was employed as an internal control to measure Nrf2 mRNA transcripts. The sequence-specific primers were as follows. The primer sequences specific for rat Nrf2 were as follows [23]: 5′-CTCTCTGGAGACGGCCATGACT-3′ (forward) and 5′CTGGGCTGGGGACAGTGGTAGT-3′ (reverse). The primers for β-actin were as follows: 5′-CCTCTATGCCAACACAGTGC-3′ (forward) and 5′CATCGTACTCCTGCTTGCTG-3′ (reverse) (GenBank accession no. NM_0311442). The relative gene expression levels were automatically computed and normalized to the reference gene β-actin, which was unaffected by the experimental circumstances, using the comparative threshold (∆∆ Ct) approach.

### 2.6. Histopathological Study

The preparation for the light microscopic analysis was as follows: The livers were left in 10% saline for three days before being dried in 70%, 90%, and 100% ethanol and cleaned with benzene. Paraffin wax was used to impregnate the samples. A rotatory microtome cut the paraffin squares into serial transverse segments with a thickness of 4 μM. An albumenized glass slide was used to mount each of the five progressive transverse paraffin sections. Hematoxylin and eosin staining was used to stain the subsequent slides obtained to illustrate the common hepatic architecture [24]. The collagen fibers were depicted using Masson’s trichrome stain [25].

### 2.7. Statistical Analysis

The statistical program Graph Prism (version 6), located in San Diego, CA, USA, was used to analyze the data. Each group’s mean ± standard deviation was used to express the results. The Tukey–Kramer multiple comparison test was used after the one-way analysis of variance (ANOVA) test to compare the means. A statistical probability of *p* < 0.05 was defined as significant.

## 3. Results

### 3.1. Biochemical Parameters

#### 3.1.1. Impact of Resveratrol on Weight

There were insignificant differences in the rats’ mean initial body weight (in grams) at the start of the experiment across the groups. At the end of the experiment, the model FED group’s body weight was significantly higher than that of the control group (*p* < 0.0001). Body weight was significantly lower in the RES + FED group than in the model FED group (*p* < 0.0001), and there were no significant differences compared with the control group (Table 1).

#### 3.1.2. Impact of Resveratrol on Liver Enzymes and Lipid Parameters

The high-fructose (FED) group exhibited increases (*p* < 0.0001) in all lipid markers (i.e., serum TAGs, liver tissue TACs, and TC) and serum liver enzyme activity (ALT and AST) compared with rats fed the standard diet (the control group). These lipid and liver markers were significantly reduced (*p* < 0.0001) in the resveratrol-treated group but still greater than those in the control group (Table 1).

#### 3.1.3. Impact of Resveratrol on Hepatic IL-6

Liver IL-6 levels were higher in rats in the FED group than those in the control group (*p* < 0.0001). The co-administration of resveratrol in group III produced a significant decrease in IL-6 levels compared with the FED group (*p* < 0.0001), with no discernible change compared with the control group (Table 1).

#### 3.1.4. Impact of Resveratrol on Hepatic AMPK, Beclin 1, and Oxidative Stress Markers

Rats in the FED group had significantly higher liver MDA than the control group (*p* < 0.0001), whereas group III’s liver MDA dropped to baseline levels when concurrently receiving resveratrol. Hepatic GSH levels and SOD activity were lowest in the FED group (*p* < 0.0001) compared to the control and RES + FED groups. The co-administration of resveratrol in group III produced significantly higher hepatic GSH levels and SOD activity than in the FED group. Nrf-2 DNA-binding activity was significantly higher in the FED group than in the control group, whereas the greatest values were seen in the RES + FED group, which significantly differed (*p* < 0.0001) from both the control and FED groups (Table 2).

AMPK and Beclin 1 levels in liver tissue were significantly lower in the FED group than in the control group (Table 2). Conversely, the RES + FED group showed the highest values, which differed from those in the FED group by a significant amount (*p* < 0.0001).

#### 3.1.5. Impact of Resveratrol on the Hepatic Expression of the Nrf-2 Gene

As shown in Table 2, the FED group’s Nrf-2 mRNA expression levels were significantly higher than those in the control group (*p* < 0.0001). Concurrently, the RES + FED group experienced a significant increase (*p* < 0.0001) compared with the normal and FED groups.

### 3.2. Histopathology

**Figure 1:**
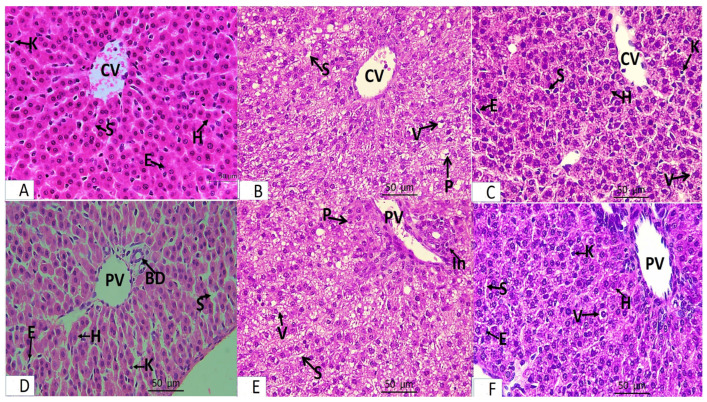
Light microscopy of liver sections from adult male rats from groups I–III. Panel (**A**,**D**) represents group I (control group). Panel (**B**,**E**) represents group II (FED group). Panel (**C**,**F**) represents group III (RES + FED group). (**A**) Hepatocytes (H) are arranged in cords radiating from the central vein (CV) and are separated by the blood sinusoids (S), which are lined by flat endothelial cells (E) and von Kupffer cells (K). (**B**) Hepatic cords are not radially arranged around the central vein (CV). They are separated by blood sinusoids (S). Most hepatocytes have vacuolated cytoplasm (V) with displaced nuclei, and some of their nuclei are small and deeply stained (P). (**C**) Anastomosing hepatic cords radiating from the central vein (CV). The blood sinusoids (S) are lined by flat endothelial cells (E) and von Kupffer cells (K). Most hepatocytes appear normal (H). Few focal areas of vacuolated hepatocytes (V). (**D**) Branches of the portal vein (PV) and the bile duct (BD) are shown. The hepatocytes (H) are separated by blood sinusoids (S), which are lined by flat endothelial cells (E) and von Kupffer cells (K). (**E**) Most hepatocytes around the portal vein (PV) have vacuolated cytoplasm (V) with displaced nuclei. Some nuclei are small and deeply stained (P). The hepatocytes are separated by blood sinusoids (S). Lymphocytic infiltration of the portal tract was observed (In). (**F**) Anastomosing hepatic cords, portal vein (PV), bile duct (BD), and blood sinusoids (S), which are lined by flat endothelial cells (E) and von Kupffer cells (K). Most hepatocytes appear normal (H). Few focal areas of vacuolated hepatocytes (V). All panels were stained with hematoxylin and eosin and originally viewed at ×400 magnification.

**Figure 2:**
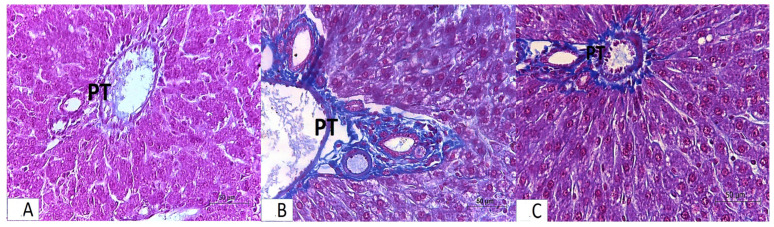
Light microscopy of liver sections of adult male rats from groups I–III. Panel (**A**) represents group I (control). Panel (**B**) represents group II (FED group). Panel (**C**) represents group III (RES + FED group). (**A**) Normal distribution of collagen fibers around the portal tract (PT). (**B**) A marked increase in collagen fiber distribution is seen around the elements of the portal tract (PT). (**C**) A mild increase in the level of collagen fiber distribution is seen around the elements of the portal tract (PT). All panels were stained with Masson’s trichrome and originally viewed at ×400 magnification.

1. Light microscopic analysis of stained liver sections (H–E) from the rats in group I (control group) revealed that the liver was composed of hepatocyte cords separated by blood sinusoids and regularly emanating from the central vein. Flat endothelial cells and von Kupffer cells lined the slit-shaped blood sinusoids. Large vesicular basophilic nuclei and granular eosinophilic cytoplasm were features of polyhedral hepatocytes (Figure 1A,D). The bile duct and portal vein branches made up the portal tract (Figure 2A). Based on Masson’s trichrome stain, the components of the portal tract and the blood sinusoidal wall were found to have a normal collagen fiber distribution (Figure 2A).
2. Examining the stained liver sections (H–E) from the rats in group II (FED group) revealed that the hepatic cords lacked the typical radial arrangement. The majority of hepatocytes had cytoplasmic vacuoles, with the nucleus displaced to the periphery. Some of the nuclei were tiny and highly stained, and the portal tracts were infiltrated by lymphocytes (Figure 1B,E). Collagen fibers were more widely distributed around the portal tract’s components according to Masson’s trichrome staining (Figure 2B).
3. Examining the stained liver sections (H–E) from the rats in group III (RES + FED group) revealed that most of the hepatic cords surrounding the central vein were in a radial pattern. Most blood sinusoids had a slit-like appearance. Granular eosinophilic cytoplasm and rounded vesicular nuclei characterized the majority of polyhedral hepatocytes. Cytoplasmic vacuoles were sparse in hepatocytes (Figure 1C,F). When the portal tract’s components were examined using Masson’s trichrome staining, the distribution of fine collagen fibers was identical to that in the control group (Figure 2C).

## 4. Discussion

The worldwide health problem of non-alcoholic fatty liver disease (NAFLD) drives up medical expenses [26]. Currently, the spotlight is focused on discovering the role played by dietary fructose in developing NASH, the most severe form of this disorder [27,28].

In this study, the mechanisms of NASH and the effects of resveratrol were investigated using a fructose-induced rat model. Fructose caused obesity in the rats, as shown by their weight gain compared with the controls, along with histopathological changes in liver specimens and increased ALT and AST levels indicative of hepatocellular damage. High triglyceride and cholesterol levels were noted in the NASH group. Similar outcomes have been found in many studies connecting reduced TAG clearance, lower VLDL export, increased hepatic lipogenesis, and decreased lipolysis with greater fat buildup in the liver [29,30].

Dietary fructose can lead to obesity and fatty liver disease, which often progresses into NASH [31]. Fructose metabolism in the liver begins with fructokinase, forming fructose-1-P. Then, aldolase-B splits it into glyceraldehyde and dihydroxyacetone phosphate, ultimately converting it into pyruvate and acetyl-CoA, increasing fatty acid synthesis and cholesterol production, and triggering more hepatic fat deposition, which leads to hepatic steatosis [32]. Fructose also stimulates the production of the carbohydrate response element-binding protein, boosting lipogenic gene expression, such as acetyl-CoA carboxylase, fatty acid synthase, and stearoyl-CoA desaturase genes [33,34].

Our data revealed that combining resveratrol with a high-fructose diet reduced liver fat and inflammation and improved the blood levels of ALT, AST, TC, and TAG and liver TAG content. These findings align with similar studies showing that resveratrol treatment lowers liver inflammation, steatosis, serum lipids, aminotransferases, insulin resistance, and cytokines [35]. Resveratrol improves lipid metabolism by downregulating SREBP-1c and upregulating AMPK and PPAR-α, leading to reduced lipogenesis and enhanced fatty acid oxidation [36]. It enhances insulin sensitivity via SIRT1 and AMPK activation and restores mitochondrial function through PGC-1α [36]. Some studies suggest that it does not affect plasma fatty acids. Factors such as food content, the length of treatment, and dosage might explain this difference [37].

The two-hit theory states that hepatic steatosis is the first hit leading to NASH, with inflammation induced by oxidative stress, cytokine activation, and toxins generated by high fructose intake as the second hit [38]. Our results show that rats with NASH had hepatic inflammation, indicated by increased IL-6 levels and histopathological analysis, and oxidative stress, shown by high MDA levels and decreased SOD activity and GSH levels. Enhanced oxidative stress in NASH is explained by (1) free fatty acid and cholesterol buildup increasing mitochondrial ROS, causing liver damage via TNF-α and IL-6; (2) high fructose intake producing ROS toxins that worsen NASH; and (3) depleted antioxidant stores by activating oxidases that increase oxidative stress [39,40].

Our research shows that restoring redox balance is key to reducing inflammation and NASH progression. Resveratrol significantly lowers liver MDA levels, increases antioxidant status, and reduces hepatic IL6 levels, these biochemical changes correlate with the histopathological improvements observed, including reduced hepatic steatosis, inflammation, and fibrosis. The attenuation of oxidative damage likely contributed to the restoration of normal liver architecture, confirming resveratrol’s anti-inflammatory and antioxidant effects in fructose-induced NASH. Resveratrol suppresses fibrosis by inhibiting TGF-β signaling and hepatic stellate cell activation and protects hepatocytes from apoptosis by regulating Bax/Bcl-2 and caspase pathways [41,42,43]. A clinical study involving individuals with NAFLD found that taking resveratrol daily for 12 weeks, in conjunction with a dietary plan and increased physical activity, improved anthropometric measures, inflammatory markers, and liver function [44]. The following study demonstrated that Nrf2, a key factor in lipid metabolism and antioxidant response, had higher gene expression and DNA-binding activity in the NASH group than in controls. As Nrf2 acts as a sensor for pro-oxidant stress, it is activated to reduce the harmful effects of ROS and improve insulin sensitivity, exhibiting anti-obesity effects [45]. The group treated with resveratrol had higher values of Nrf2 than the control and model groups; this finding aligns with the observed histological improvements, such as decreased cytoplasmic vacuolization and normalized collagen distribution, as indicated in the Masson’s trichrome staining. A related study showed that polydatin, a natural precursor of resveratrol, activates Nrf2 to protect against NASH; however, their study did not explore Nrf2 DNA-binding activity as a molecular marker. In contrast, we provide direct evidence of increased Nrf2 DNA-binding activity, highlighting its critical role in oxidative stress modulation [46]. Resveratrol changes KEAP1 cysteine sulfhydryl groups, preventing KEAP1–NRF2 interaction, allowing Nrf2 to enter the nucleus, activating antioxidant genes, and combating inflammation and stress. It also triggers epigenetic alteration of the Nrf2 gene for better oxidative defense [47,48]. These findings collectively indicate that Nrf2 is involved in NASH and can be targeted for fatty liver disease treatment.

**Figure 3:**
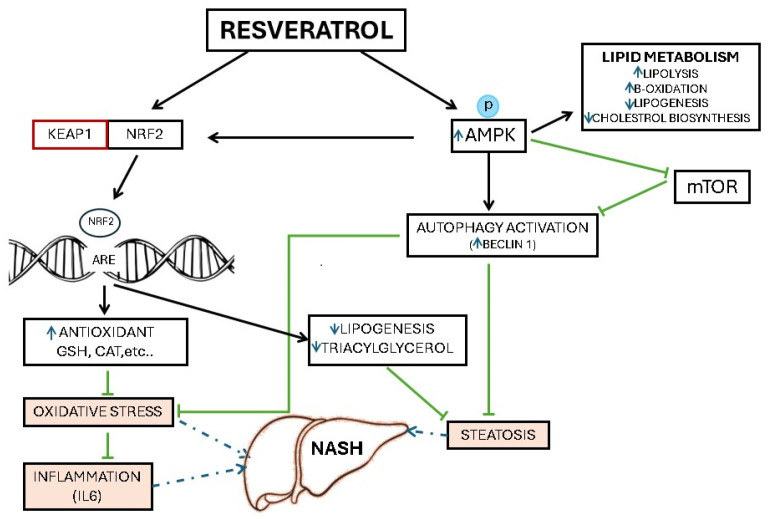
Schematic summary of the proposed protective roles of resveratrol through the induction of AMPK/Nrf2 in non-alcoholic steatohepatitis. The classical understanding is that Nrf2 coordinates the elimination of ROS and electrophiles derived from lipid peroxidation, thus preventing hepatocellular oxidative stress and mitochondrial dysfunction. In addition, there is growing evidence in the literature that Nrf2 regulates fatty acid metabolism by repressing genes that promote lipid accumulation in hepatocytes. AMPK induction by resveratrol also activates autophagy that lowers hepatic lipid load via lipophagy, eliminates dysfunctional mitochondria, and hence reduces the ROS level. *Resveratrol* → *KEAP1*: Inhibits KEAP1, activating NRf2. *KEAP1* → *NRf2*: KEAP1 suppresses NRf2; Resveratrol removes this suppression. *NRf2* → *Antioxidant*: Increases antioxidant gene expression. *Antioxidant* → *Oxidative Stress*: Antioxidants reduce oxidative stress. *Oxidative Stress* → *NASH*: Promotes liver damage leading to NASH. *Oxidative Stress* → *Inflammation*: Stimulates inflammatory cytokines like IL-6. *Inflammation* → *NASH:* Chronic inflammation worsens NASH. *Resveratrol* → *AMPK*: Activates AMPK, regulating energy metabolism. *AMPK* → *Lipid Metabolism*: Enhances lipolysis, reduces lipid and cholesterol. *AMPK* → *mTOR:* Suppresses mTOR, reducing lipid synthesis. *AMPK* → *Autophagy*: Promotes autophagy, reducing lipid accumulation. *Autophagy* → *Lipogenesis*: Decreases lipogenesis and triacylglycerol. *Lipogenesis* → *Steatosis*: Excess lipids lead to steatosis. *Steatosis* → *NASH*: Steatosis progresses to NASH with inflammation.

Maintaining equilibrium in cells and preventing stress through autophagy is crucial for maintaining cell quality. Beclin 1, which regulates autophagy, markedly dropped in the NASH group compared with the controls and improved in the resveratrol-treated group. This conclusion is corroborated by additional research showing that autophagy is suppressed in animal models of NASH and that its restoration may decrease the progression of NAFLD [49,50,51]. Resveratrol induces autophagy through AMPK-mediated activation of transcription factor EB (TFEB). This promotes the formation and fusion of autophagosomes and lysosomes into autophagic lysosomes [52]. In addition, resveratrol also inhibits the activity of the NLRP3 inflammasome and upregulates the expression of the AMPK-SIRT1 signaling pathway to reduce key proteins of the MAPK signaling pathway and ultimately induce autophagy [53,54]. Autophagy helps protect the liver by reducing triglyceride and cholesterol buildup, improving insulin signaling, blocking TNFα and Fas-death receptor-mediated liver injury, activating Nrf2, and decreasing endoplasmic reticulum stress [55]. However, while autophagy protects hepatocytes, too much of it can lead to autophagic cell death, which worsens possible liver damage (Figure 3) [56,57].

Hepatic AMPK levels decreased in the NASH group, even with low ATP levels from fructose metabolism, indicating that other factors regulate AMPK activity during NASH. Inflammatory substances such as TNFα and IL6 may impair AMPK activation [58]. A further theory suggests that spikes in fructose in the portal vein cause uncontrolled triose production, causing methylglyoxal, which creates carbonyl stress on the arginine in AMPK’s c subunits, blocking the enzyme. These mechanisms explain why fructose’s effects align with AMPK inactivation despite increasing AMP, which triggers AMPK activation [59]. A recent study demonstrated that resveratrol could lower oxidative stress and cardiac ischemia/reperfusion injury via AMPK/p38/Nrf2 pathway activation [60], but its specific role in fructose-induced NASH models has not been fully elucidated. Our data revealed a significant improvement in AMPK phosphorylation, linking it to improved lipid metabolism, reduced inflammation, Nrf2 pathway activation, and enhanced autophagy. AMPK responds to resveratrol by increasing its activity, lowering energy use, and reducing energy loss. This leads to enhanced beta-oxidation, glucose transport, and glycolysis, decreased cholesterol synthesis, and reduced lipogenesis, shifting cells to produce ATP. Activated AMPK also boosts Nrf2/HO-1 signaling and suppresses mTOR pathways, promoting autophagy and mitochondrial biogenesis, which helps improve NASH (Figure 3) [61,62].

## 5. Conclusions

This study highlights the novelty of integrating oxidative stress, autophagy, and inflammation under the regulation of the AMPK/Nrf2 signaling axis in a fructose-induced NASH model. Our findings demonstrate that resveratrol enhances Nrf2 DNA-binding activity, restores phosphorylated AMPK levels, and modulates key markers such as Beclin 1 and IL-6. This molecular perspective bridges critical gaps in understanding the mechanisms underlying fructose-induced NASH and suggests that resveratrol could serve as a promising adjunctive therapeutic agent in managing NASH and exploring targeted therapeutic strategies. Further studies are warranted to explore its efficacy in human clinical trials, optimize dosing regimens, and investigate its potential synergistic effects when combined with existing treatments.

Study Limitations:

1. The parameters for liver morphology and weights were not measured
2. PPARα, CPT1α, and ACOX1 expression levels were not measured.

## Figures and Tables

**Table 1:** Impact of resveratrol on liver IL-6 and some metabolic markers.

Group	Group I	Group II	Group III	ANOVA	
Variable				F-Value	*p*-Value
Initial body weight (grams)	194.7 ± 4.87	195.55 ± 4.51	196.3 ± 3.81	0.655	0.5233
Final body weight (grams)	264.33 ± 15.4	315.3 ± 11.1 ^a^	265.4 ± 12.4 ^b^	94.18	<0.0001 *
Triacylglycerol levels (mg/dL)	79.253 ± 2.9	212.56 ± 13.1 ^a^	96.23 ± 3.2 ^a,b^	1646.1	<0.0001 *
Total cholesterol levels (mg/dL)	91.47 ± 6.1	182.95 ± 12.1 ^a^	98.57 ± 5.26 ^a,b^	727.2	<0.0001 *
AST (U/L)	28.27 ± 0.54	91.91 ± 7.2 ^a^	42.26± 1.2 ^a,b^	1247.3	<0.0001 *
ALT (U/L)	24.54 ± 0.16	57.56 ± 7.81 ^a^	29.35 ± 1.7 ^a,b^	298.6	<0.0001 *
Liver tissue Triacylglycerol levels (mg TAG/mg protein/mL)	0.265 ± 0.05	0.48 ± 0.103 ^a^	0.348 ± 0.02 ^a,b^	50.38	<0.0001 *
IL-6 levels (pg/mL)	80.31 ± 16.5	129.7 ± 33.9 ^a^	87.3 ± 21.9 ^b^	22.46	<0.0001 *

Means ± SD are used to express values. * *p* was deemed significant at <0.05; Tukey’s test was used for multiple comparisons after one-way ANOVA. ^a^—Significant compared with the control group; ^b^—significant compared with the FED group. IL-6—interleukin-6; ALT—alanine aminotransferase; AST—aspartate aminotransferase.

**Table 2:** Impact of resveratrol on oxidative stress markers, Beclin 1, and AMPK.

Group	Group I	Group II	Group III	ANOVA	
Variable				F-Value	*p*-Value
MDA (nmol/mg protein/mL)	63.59 ± 14.5	202.93 ± 72.7 ^a^	74.62 ± 21.3 ^b^	60.46	<0.0001 *
SOD units/mg protein	422.65 ± 75.06	269.8 ± 62.7 ^a^	446.93 ± 55.4 ^b^	43.76	<0.0001 *
GSH (mg/g liver tissue)	1.216 ± 0.19	0.86 ± 0.14 ^a^	1.11 ± 0.32 ^b^	11.76	<0.0001 *
DNA-binding activity of Nrf2	0.436 ± 0.02	0.53 ± 0.01 ^a^	0.64 ± 0.02 ^a,b^	516.31	<0.0001 *
Nrf2 mRNA relative expression	1.063 ± 0.36	1.49 ± 0.08 ^a^	1.80 ± 0.34 ^a,b^	32.1731	<0.0001 *
Beclin 1 (ng/mg protein/mL)	1.06 ± 0.10	0.82 ± 0.26 ^a^	1.17 ± 0.12 ^b^	19.59	<0.0001 *
Phosphorylated active form of AMPK level (ng/mg protein/mL)	1.86 ± 0.3	1.50 ± 0.35 ^a^	2.07 ± 0.39 ^b^	13.60	<0.0001 *

Means ± SD are used to express values. * *p* was deemed significant at <0.05; Tukey’s test was used for multiple comparisons after one-way ANOVA. ^a^— Significant compared with the control group; ^b^—significant compared with the FED group. SOD—superoxide dismutase; GSH—glutathione; MDA—malondialdehyde; Nrf2—nuclear factor-erythroid 2-related factor.

## Data Availability

All data supporting the findings of this study are available upon reasonable request.
